# Attitudes Towards the Use of Artificial Intelligence in Healthcare: A Conjoint Analysis Survey in Singapore

**DOI:** 10.1007/s41649-025-00380-2

**Published:** 2025-11-15

**Authors:** Hui Jin Toh, Hui Yun Chan, Thomas Ploug, Søren Holm, Tamra Lysaght

**Affiliations:** 1 Centre for Biomedical Ethics, Yong Loo Lin School of Medicine, National University of Singapore, Singapore; 2 Centre for AI Ethics, Law, and Policy, Aalborg University, Aalborg, Denmark; 3 School of Health and Welfare, Halmstad University, Halmstad, Sweden; 4 Centre for Social Ethics and Policy, Department of Law, School of Social Sciences, University of Manchester, Manchester, UK; 5 Centre for Medical Ethics, HELSAM, Faculty of Medicine, University of Oslo, Oslo, Norway; 6 Sydney Health Ethics, Sydney School of Public Health, Faculty of Medicine and Health, University of Sydney, Sydney, Australia

**Keywords:** AI ethics, Artificial intelligence, Attitudinal research, Fairness, Transparency, Moral responsibility, AI in healthcare

## Abstract

Novel artificial intelligence (AI) is increasingly being used as a clinical decision support tool in healthcare. Despite AI’s growing use and improved quality in clinical decision-making, questions persist about potential harms and the lack of transparency in their algorithms. Implementation of AI technologies in healthcare must align with local norms and ethical standards if the purported benefits are to be achieved in specific contexts. Using choice-based conjoint analysis, we examined how Singaporeans evaluate different principles related to AI decision-making in healthcare. Six attributes were included: decision type, severity, explainability, quality, responsibility, and discrimination. Among 596 respondents, 51% reported fear that AI would unintentionally harm humans, while 87% feared increased surveillance. Responsibility had the highest relative importance (31.5%) for AI use in healthcare, followed by explainability (27.7%) and discrimination (15.9%). The most valued attribute levels were AI recommendations being as explainable as doctors’, doctors retaining responsibility for treatment decisions, and AI systems being tested for discrimination. Our respondents showed higher levels of trust, hope, and fear toward AI, with a stronger preference for explainability over doctor responsibility. While having AI outperform doctors in generating clinical suggestions is desirable, principles such as explainability, human oversight, and fairness are more important for the people whose lives AI will impact.

## Introduction

With growing accessibility of healthcare data and advancements in analytics, artificial intelligence (AI) presents promising prospects to revolutionise health care. AI technologies can now improve the accuracy and efficiency of drug treatments, improve patient outcomes, and potentially reduce the economic burden on healthcare systems (Rahman et al. 2024). Currently, novel AI systems using large language models (LLMs) are employed in various aspects of health care. Some examples are ClinicalBERT (Huang et al. 2019) that predicts hospital readmission risk based on unstructured clinical text data in electronic health records, PathChat (Lu et al. 2024) that is used as clinical decision support (CDS) to analyse histopathology images and suggest diagnosis, and algorithms such as deep neural networks (DNNs) (Paul et al. 2020) to do virtual drug screening to speed up drug discovery and development.

Despite its potential for health care, concerns have been raised about the lack of transparency and explainability of AI algorithms. The opacity could lead to harms, including misdiagnoses and adverse patient outcomes as well as exacerbating racial and gender inequalities stemming from algorithmic bias and lack of diversity in training data (Norori et al. 2021; Amann et al. 2020; Weiss et al. 2018). There are also concerns about establishing responsibility and legal liability for when AI systems are used in error and cause harms. These concerns have prompted extensive research on explainable AI techniques, including methods for interpreting deep learning models in clinical contexts, developing more transparent algorithms for clinical decision support, and creating human-understandable explanations of AI-driven diagnoses (Obermeyer et al. 2019; Allen 2024). However, these studies are still in their infancy, and further validation and development of the techniques are still needed (Obermeyer et al. 2019; Allen 2024).

The World Health Organisation (WHO)’s 2021 report on Ethics and Governance of Artificial Intelligence for Health provides guidelines for the responsible development, design, use, and regulation of AI. The report’s guiding principles stress that AI should be used, governed, and regulated in ways that promote autonomy, well-being, trust, accountability, and equity, while ensuring sustainability. Recognising the potential harms that AI applications may pose as they are implemented rapidly in practice (Coiera et al. 2023; Robles and Mallinson 2025), the WHO published a list of updated key regulatory guidelines in 2023 (WHO 2023a, b). These guidelines aimed to foster the safe and effective use of AI in health care globally. In addition, other principle-based governance frameworks (Reddy et al. 2020; Hassan et al. 2024) have been proposed in recent years for the responsible use of AI in healthcare, to practically address the concerns on the ethical and regulatory aspects.

While generating guiding principles to meet legal and ethical obligations is crucial, principle-based approaches tend to be abstract and broad and often do not fully address the ethical complexities and nuances inherent in AI applications in practice within local contexts. Understanding public values is key to identifying these nuances and making informed trade-offs (Frost et al. 2024; Wilson 2022; Banerjee et al. 2022; Stahl 2021; Bélisle-Pipon et al. 2023).

To ensure that implementation of AI systems in practice is for the public good, AI systems must be sensitive to ethical and social norms across diverse multicultural contexts. Aligning healthcare AI with public values and gaining public confidence is critical, as any significant failure with AI could greatly diminish trust in the healthcare system. The importance of incorporating public attitudes and values into the governance of AI technologies in building and maintaining trust is widely recognised (Frost et al. 2024; Wilson 2022; Banerjee et al. 2022; Stahl 2021; Bélisle-Pipon et al. 2023). Understanding public values allows AI developers and policymakers to align AI technologies with accepted ethical norms while optimising benefits (Robles and Mallinson 2025). Moreover, as AI’s training algorithms require access to large health care datasets that have been built with public funding and resources, engaging with the communities who contribute to those datasets can maximise fairness and inclusion in the ethical governance of AI in healthcare (Norori et al. 2021; von Siemens 2024). However, despite the recognised importance of understanding public values related to AI governance, there remains limited empirical research in the Southeast Asian healthcare context. This study aimed to survey public attitudes towards ethical AI governance in the Southeast Asian health economy of Singapore.

## Singapore's Approach to Healthcare AI Integration

Singapore is a leader in integrating AI into health care practices. As part of Singapore’s ongoing national innovation strategy (National Research Foundation, Prime Minister's Office, Singapore 2025) to proactively use AI to personalise healthcare, the government introduced the National AI Strategy in 2019. By developing tools for CDS, the National AI Strategy aims to use AI to predict and manage chronic diseases for patient self-care. In line with this initiative were the AI in healthcare guidelines (AIHGle) (Ministry of Health, Health Sciences Authority, and Synapxe 2021) developed by the Singapore Ministry of Health. These guidelines outline important guiding principles (i.e., fairness, responsibility, transparency and explainability, and patient-centricity) for developers and implementers of AI in healthcare. This model aims to balance innovation and public trust in AI (Yeong 2020).

Empirical studies on the ethical use of AI in healthcare settings are concentrated in the United States (US) (Hesso et al. 2023; Turchioe et al. 2023; Khullar et al. 2021) and the United Kingdom (UK) (King et al. 2023; Benda et al. 2024). A US survey exploring potential benefits and risks of using AI for decision-making identified key risks as the nature of diagnostic tasks, lack of transparency in the AI process, safety concerns around AI-driven recommendations, and complexities in interpreting results (Esmaeilzadeh 2020). A recent meta-analysis (Wu et al. 2023) of qualitative studies across countries including China, India, US, Germany, Canada, Belgium, and the Netherlands primarily found concerns about AI’s opacity leading to public distrust and who should be held accountable for errors made by AI CDS. Reviews synthesising public surveys primarily conducted in the US and European countries (including the UK, Germany, France, Italy, and the Netherlands) (Scott et al. 2021; Young et al. 2021; Yang et al. 2022; Hogg et al. 2023; Tang et al. 2023) have reported positive attitudes in the potential of AI to enhance diagnostic accuracy, improve care efficiency and speed, and improve healthcare access in underserved areas. Nonetheless, there remain concerns about physician oversight, accountability, minimising harms, and safeguarding empathy as a core element in healthcare, even with the growing presence of AI (Scott et al. 2021; Young et al. 2021; Yang et al. 2022; Hogg et al. 2023; Tang et al. 2023). These studies, however, typically employed conventional survey methods that did not require respondents to make explicit trade-offs between competing values and priorities. It is therefore challenging to determine which values take precedence over others when developing practical policy guidelines for AI in healthcare.

A Danish survey (Ploug et al. 2021) addressed this limitation by examining how the general public prioritise factors influencing AI decision-making in healthcare, using a choice-based experiment called conjoint analysis. Conjoint analysis is a statistical method used in surveys to determine how respondents make decisions based on hypothetical scenarios, with each scenario containing several competing attributes that contribute to their decision-making (Huber 2005). Conjoint analysis operates on the premise that individuals prioritise each attribute differently and make trade-offs between them. This survey (Ploug et al. 2021) found that the most important consideration was the physician retaining the final responsibility for treatment decisions, followed by explainability and whether the system has been tested for discrimination.

To date, Singapore lacks well-defined, culturally applicable evidence on public acceptance and attitudes towards the use of AI to augment CDS. We aimed to examine how Singaporeans prioritise various dimensions of AI-augmented decision-making in Singapore using a choice-based conjoint analysis. This cross-sectional study adapted the survey instrument developed by Ploug et al. (2021) for use in the Singapore population. We chose this instrument because of its ability to capture how individuals weigh multiple competing factors in healthcare AI decision-making—providing a deeper understanding of what is important to Singaporeans than conventional surveys. The results also provide insights for developing culturally informed frameworks for responsible AI implementation in Singapore’s healthcare system. Results from this study were compared with Ploug et al. (2021)’s to identify any socio-cultural nuances influencing AI decision-making preferences in Singapore’s healthcare context. We also examined any associations of the results of this conjoint analysis with respondent demographics.

## Methods

### Survey Recruitment

A choice-based conjoint (CBC) analysis survey was administered to the Singapore Health Opinion Population Survey (HOPS) panel (2024) from August to October 2023. The HOPS panel is an online survey panel consisting of 2527 members and is hosted at the Centre for Biomedical Ethics, National University of Singapore. The panel’s operations are approved by the National University of Singapore Institutional Review Board to be used for public health and ethics studies (IRB ref: LH-18–011). Email invitations were sent to 1274 members selected at random (approximately half of the panel), and if we did not receive a response by days 7, 14, and 28, reminder emails were sent to members who had not completed the survey. Respondents received an SGD 10 supermarket electronic voucher.

### Survey Instrument

The survey instrument was adapted from Ploug et al. (2021) and pilot tested prior to administration to the HOPS sample. The full survey instrument is available in the supplementary materials. Respondents were tasked with choosing their preferences for one out of three hypothetical AI systems if they were to be admitted to a hospital for diagnosis and treatment.

**Fig. 1:**
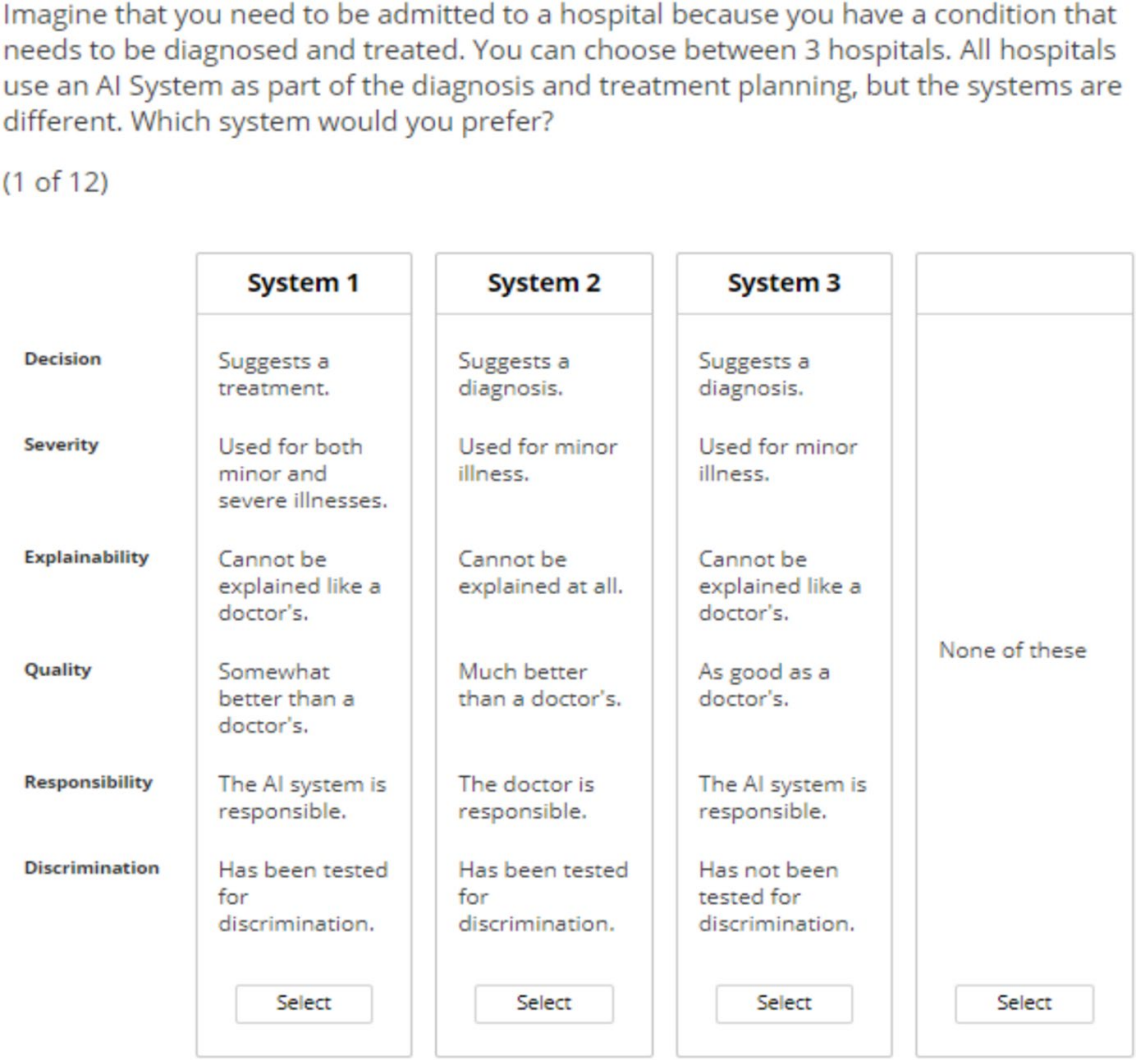
An example hypothetical scenario where respondents were asked to select their most preferred AI system as a CDS for diagnosis and treatment of their condition

The survey was administered in Lighthouse Studio 9.15.9 (Sawtooth Pty Ltd, USA). Table 1 shows the six attributes and the respective attribute levels that were used for this conjoint analysis, and Fig. 1 depicts an example of a choice-based scenario. The survey also consisted of a section containing 5-point Likert scale questions on trust in health care and technology and opinions about AI and a section on demographics that includes age, ethnicity, gender, highest education level, chronic conditions (if any), number of visits to the family doctor in the last year, and number of hospitalisations in the last year.

**Table 1:** Attributes and their definitions, and attribute levels of the choice-based conjoint analysis on the use of AI as a CDS in healthcare

Attribute	Attribute levels
**Decision** indicates what the AI is used for	1. Suggests a diagnosis 2. Suggests a treatment
**Severity** indicates the severity of illnesses the AI system is used for	1. Used for minor illness 2. Used for both minor and severe illnesses
**Explainability** indicates how well someone understands and can explain the AI system’s suggestions	1. Can be explained like a doctor’s 2. Cannot be explained like a doctor’s 3. Cannot be explained at all
**Quality** a measure of how well the AI system is able to provide suggestions	1. Much better than a doctor’s 2. Somewhat better than a doctor’s 3. As good as a doctor’s
**Responsibility** is about who ultimately bears responsibility for the AI system's suggestions	1. The doctor is responsible 2. The AI system is responsible
**Discrimination** indicates whether the AI system has been tested for whether it unfairly discriminates against certain patient groups	1. Has been tested for discrimination 2. Has not been tested for discrimination

### Statistical Analysis

Part-worth utilities and relative attribute importance were calculated using hierarchical Bayes analysis in Lighthouse Studio (Sawtooth Pty Ltd, Utah, USA), where higher utility values indicated stronger preferences for specific attribute levels. The relative importance of attributes was determined by converting utility value ranges into percentages that summed to 100%. Statistical analysis was performed using IBM SPSS Statistics 29, including descriptive statistics for demographic data and univariate analysis to examine associations between respondent characteristics and importance percentages, as well as trust, fear, and hope scales. Relationships between utilities and these scales were assessed using correlation analysis with Bonferroni correction, while chi-square tests, Student’s t-test, and one-way ANOVA were used for categorical, continuous, and multiple group comparisons, respectively, with statistical significance set at *p* < 0.05. To ensure representativeness, the survey data were weighted according to Singapore’s population ethnicity distribution (74.3% Chinese, 13.5% Malay, 9% Indian, and 3.2% others) due to underrepresentation of Malays and Indians in the HOPS panel. The more detailed description of the statistical analysis is available in the supplementary materials.

### Ethics Oversight

Exemption from ethics review for this study was granted by the National University of Singapore Institutional Review Board (Reference no**.** LH-18–011).

## Results

### Respondent Characteristics

In the period from August to October 2023, this survey was distributed to 1273 randomly selected members of the HOPS panel, and of these, 596 respondents completed the survey (response rate = 47%). The respondent characteristics are presented in Table 2. Of the respondents, 59% were female, and their age ranged from 21 to 83 years, with a mean age of 50 years (SD = 13.8). In terms of ethnicity, 517 (87%) were Chinese, 47 (8%) Indians, and 17 (3%) Malays. Conjoint analysis was conducted on data that were weighted for ethnicity so that the respondents’ data can be compared to those of the Singapore Census.

**Table 2:** Respondent characteristics

Respondent characteristic	No. of respondents (%)
Age group (years)	
21–30	57 (10%)
31–40	116 (19%)
41–50	144 (24%)
51–60	117 (20%)
61–70	129 (22%)
71–80	32 (5%)
81–90	1 (1%)
Gender	
Female	315 (59%)
Male	245 (41%)
Ethnicity	
Chinese	517 (87%)
Malay	17 (3%)
Indian	47 (8%)
Others	15 (3%)
Highest education level	
No formal education	2 (1%)
Pre-primary	0 (0%)
Primary	4 (1%)
Secondary	93 (16%)
Post-secondary	28 (5%)
Diploma	127 (22%)
A Levels	22 (4%)
University—Bachelor’s degree	239 (40%)
University—Postgraduate degree	81 (14%)
Visited family doctor last year	402 (67%)
Median no. of visits to a family doctor in the last year	3
Chronic illness	139 (23%)
Hospitalised last year	47 (8%)
Median no. of hospitalisations in the last year	1

### Trust, Fear, and Hope regarding AI in Healthcare

Table 3 summarises responses related to trust in healthcare and technology, as well as fear and hope about AI. Most of the respondents reported some or a lot of trust in physicians (95%), in the health care system (83%), and in technology (88%). When asked about the level of fear about AI, slightly more than half (58%) had some or a lot of fear that AI will lead to unemployment, while 51% had some or a lot of fear that AI will cause unintentional harm to humans. A large majority (87%) of the respondents had some or a lot of fear that AI would lead to increased data collection and mass surveillance. Regarding the topic of hope for AI, 62% had some or a lot of hope that AI will lead to more quality of life, while the opinions on the belief that AI will lead to peace and political stability were nearly evenly split.

**Table 3:** Trust, fear, and hope in AI

Statement	None/not at all	Very little	Little	Some/somewhat	A lot/certainly	Don’t know	Mean (SD)
Trust in healthcare and technology							
I have trust in the health care system	4 (1%)	31 (5%)	49 (8%)	305 (51%)	192 (32%)	15 (3%)	4.17 (0.86)
I have trust in physicians	2 (1%)	5 (1%)	20 (3%)	267 (45%)	296 (50%)	6 (1%)	4.46 (0.65)
I have trust in technology	0 (0%)	10 (2%)	48 (8%)	376 (63%)	151 (25%)	11 (2%)	4.18 (0.67)
Fears about AI							
I believe that AI will lead to unemployment	28 (5%)	66 (11%)	114 (19%)	229 (38%)	119 (20%)	40 (7%)	3.78 (1.22)
I believe that AI will cause unintentional harm to humans	15 (3%)	66 (11%)	141 (24%)	213 (36%)	89 (15%)	72 (12%)	3.86 (1.23)
I believe that AI will lead to loss of control to machines	35 (6%)	84 (14%)	131 (22%)	198 (33%)	71 (12%)	77 (13%)	3.70 (1.36)
I believe that AI will lead to increased data collection and mass surveillance	5 (1%)	14 (2%)	30 (5%)	174 (29%)	347 (58%)	26 (4%)	4.55 (0.82)
Hope about AI							
I believe that AI will lead to more jobs	48 (8%)	66 (11%)	153 (26%)	213 (36%)	42 (7%)	74 (12%)	3.60 (1.35)
I believe that AI will lead to longer lives	38 (6%)	42 (7%)	109 (18%)	200 (34%)	81 (14%)	126 (21%)	4.04 (1.42)
I believe that AI will lead to more quality of life	18 (3%)	37 (6%)	95 (16%)	254 (43%)	117 (19%)	75 (14%)	4.07 (1.17)
I believe that AI will lead to peace and political stability	86 (14%)	88 (15%)	114 (19%)	133 (22%)	28 (5%)	147 (25%)	3.62 (1.72)

The mean score was calculated based on the options “none/not at all” = 1, “very little” = 2, “little” = 3, “some” = 4, and “a lot/certainly “ = 5. The “don’t know” option was excluded from the calculation of the mean score.

### Relative Importance of Attributes of AI System

**Fig. 2:**
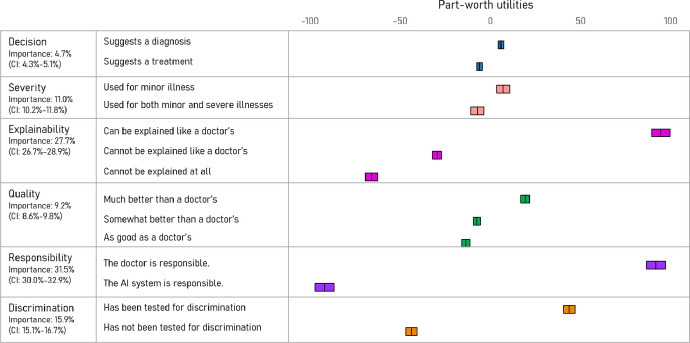
Relative importance of attributes and part-worth utilities of attribute levels from the CBC analysis. A higher utility value represents a greater preference of the attribute level. The width of the boxes corresponds with 95% confidence intervals

As presented in Fig. 2, respondents attributed the highest value to responsibility (relative importance = 31.5%), followed by explainability (27.7%) and discrimination (15.9%). Severity and decision were relatively less important in the respondents’ decision-making on a desired AI system in a hospital setting. The average part-worth utilities were the highest for the following three attributes: (i) suggestions made by AI can be explained like a doctor’s, (ii) the responsibility for treatment decisions being placed on the doctor, and (iii) the AI system having tested for discrimination.

### Relationship between Respondent Demographics and Relative Importance of Attributes, and Opinions about AI

Table 4 outlines the results from our subgroup analyses. There were no statistically significant differences or strong correlations in importance percentages in relation to the following variables: gender, ethnicity, chronic diseases, visit to the family doctor in the last year, and hospitalisation in the last year. In terms of highest education attainment, those with a bachelor’s degree tend to prioritise responsibility over those without a degree (importance = 33.1% versus 29.6%, *P* = 0.016). Our analysis results also identified a weak but statistically significant negative correlation between age and preference for explainability (*R* = − 0.088, *p* < 0.05). As respondent age increased, the relative importance placed on the explainability of AI systems slightly decreased. No strong correlations were found between relative importance and opinions about AI (i.e., the trust, fear, and hope scales). In addition, as indicated by Table 4, the only statistically significant finding was the weak positive correlation between the fear scale and the quality attribute (*R* = 0.018).

**Table 4:** Relative importance of attributes by age, gender, ethnicity, highest education level, chronic disease, visit to the family doctor in the last year, hospitalisation in the last year, trust scale, fear scale, and hope scale. CI corresponds to 95% confidence intervals (95%CI)

Respondent characteristic	Relative importance of attributes					
	Decision	Severity	Explanation	Quality	Responsibility	Discrimination
Age	*R* = − 0.011	*R* = − 0.032	*R* = − 0.088*	*R* = − 0.021	*R* = 0.056	*R* = 0.075
Gender						
Female (*N* = 245)	4.8% (CI: 4.3–5.1%)	11.5% (CI: 10.4–12.7%)	28.4% (CI: 26.6–30.1%)	9.1% (CI: 8.1–10.1%)	30.4% (CI: 28.1–32.7%)	15.8% (CI: 14.5–17.1%)
Male (*N* = 351)	4.6% (CI: 4.3–5.3%)	10.3% (CI: 9.1–11.4%)	26.9% (CI: 25.1–28.6%)	9.3% (CI: 8.3–10.3%)	33.0% (CI: 30.7–35.3%)	16.0% (CI: 14.7–17.3%)
*P*-value	0.541	0.120	0.196	0.752	0.084	0.815
Ethnicity						
Chinese (*N* = 517)	4.7% (CI: 4.3–5.1%)	10.9% (CI: 10.1–11.8%)	28.0% (CI: 26.8–29.2%)	9.0% (CI: 8.4–9.8%)	31.4% (CI: 29.9–32.9%)	16.0% (CI: 15.1–16.8%)
Malay (*N* = 17)	7.6% (CI: 4.4–10.9%)	12.4% (CI: 5.1–19.6%)	23.1% (CI: 14.4–31.7%)	8.3% (CI: 4.7–11.9%)	34.1% (CI: 23.0–45.3%)	14.5% (CI: 9.5–19.5%)
Indian (*N* = 47)	4.3% (CI: 3.2–5.5%)	12.6% (CI: 9.4–15.8%)	25.1% (CI: 21.5–28.8%)	10.1% (CI: 7.9–12.2%)	33.2% (CI: 27.6–38.9%)	14.6% (CI: 12.2–17.0%)
Others (*N* = 15)	4.2% (CI: 1.4–6.9%)	7.3% (CI: 3.8–10.9%)	32.4% (CI: 24.8–39.9%)	12.3% (CI: 6.3–18.2%)	25.7% (CI: 17.4–34.0%)	18.2% (CI: 11.1–25.4%)
*P*-value	0.059	0.318	0.143	0.316	0.493	0.587
Highest education level						
No bachelor’s degree (*N* = 276)	4.7% (CI: 4.2–5.2%)	11.8% (CI: 10.6–13.0%)	28.2% (CI: 26.6–29.8%)	9.4% (CI: 8.5–10.2%)	29.6% (CI: 27.5–31.6%)	16.4% (CI: 15.1–17.6%)
Has a bachelor’s degree (*N* = 320)	4.8% (CI: 4.2–5.3%)	10.4% (CI: 9.3–11.4%)	27.3% (CI: 25.8–28.9%)	9.0% (CI: 8.1–9.9%)	33.1% (CI: 31.1–35.1%)	15.5% (CI: 14.4–16.5%)
*P*–value	0.879	0.085	0.435	0.535	0.016	0.274
Chronic disease						
No chronic disease (*N* = 457)	4.6% (CI: 4.2–5.0%)	10.9% (CI: 10.0–11.8%)	28.3% (CI: 27.0–29.6%)	9.1% (CI: 8.4–9.8%)	31.2% (CI: 29.6–32.9%)	15.9% (CI: 14.9–16.8%)
Has chronic disease (*N* = 139)	5.2% (CI: 4.4–5.9%)	11.3% (CI: 9.5–13.2%)	25.9% (CI: 23.7–28.0%)	9.4% (CI: 8.0–10.8%)	32.3% (CI: 29.3–35.4%)	15.9% (CI: 14.3–17.6%)
*P*-value	0.182	0.690	0.056	0.708	0.531	0.949
Visits to the family doctor in the last year						
Visited family doctor (*N* = 402)	4.6% (CI: 4.2–5.0%)	11.8% (CI: 8.5–15.1%)	27.4% (CI: 26.1–28.7%)	8.9% (CI: 8.2–9.5%)	31.4% (CI: 29.6–33.1%)	16.4% (CI: 15.4–17.3%)
Did not visit family doctor (*N* = 192)	5.0% (CI: 4.3–5.7%)	10.9% (CI: 10.1–11.8%)	28.4% (CI: 26.4–30.5%)	7.1% (CI: 5.9–8.3%)	31.7% (CI: 29.2–34.3%)	14.9% (CI: 13.5–16.2%)
*P*-value	0.317	0.118	0.410	0.170	0.810	0.080
Hospitalisation in the last year						
Hospitalised (*N* = 47)	4.4% (CI: 3.3–5.5%)	11.8% (CI: 8.5–15.1%)	28.4% (CI: 24.0–32.8%)	11.0% (CI: 8.3–13.7%)	28.2% (CI: 22.2–34.3%)	16.3% (CI: 13.2–19.3%)
Not hospitalised (*N* = 549)	4.8% (CI: 4.4–5.2%)	10.9% (CI: 10.1–11.8%)	27.7% (CI: 26.5–28.8%)	9.0% (CI: 8.4–9.6%)	31.8% (CI: 30.3–33.2%)	15.8% (CI: 15.0–16.7%)
*P*-value	0.514	0.625	0.764	0.170	0.267	0.949
Trust, fear, and hope scales regarding AI						
Trust scale	*R* = − 0.027	*R* = 0.004	*R* = − 0.036	*R* = –0.065	*R* = 0.049	*R* = 0.015
Fear scale	*R* = 0.048	*R* = − 0.018	*R* = − 0.040	*R* = 0.081*	*R* = − 0.049	*R* = 0.039
Hope scale	*R* = 0.029	*R* = 0.040	*R* = − 0.041	*R* = 0.002	*R* = 0.027	*R* = − 0.016

Gender, ethnicity, highest education level, chronic disease, visit to the family doctor in the last year, and hospitalisation in the last year were treated as categorical variables. A *p*-value of < 0.05 is considered statistically significant. Age and trust, fear, and hope scales were treated as continuous variables. Correlation analysis (Spearman’s correlation) was performed to examine the association between these variables and the sociodemographic variables. Strong, moderate, and weak correlations are defined as *R* ≥ 0.50, *R* = 0.30–0.49, and *R* = 0.20–0.29, respectively

^**^Correlation is significant at the 0.01 level (2-tailed)

^*^Correlation is significant at the 0.05 level (2-tailed)

## Discussion

This study examined how different competing values were prioritised when using AI to support clinical decision-making in Singapore, as well as levels of trust, fears, and hopes about AI technology. Our main findings were similar to Ploug et al. (2021)’s survey, with responsibility being the most important value, followed by explainability, discrimination, and quality. Similarly, in both studies, the average part-worth utilities were the highest for the following dimensions: the doctor being responsible for the AI’s suggestions, the AI suggestions being explainable like a doctor’s, and the AI system having been tested for discrimination. However, in our study, there was a stronger preference for explainability rather than the doctor being held responsible for the AI’s recommendations. That is, while doctors taking responsibility for the AI is relatively more important, the highest value was placed on the ability to explain the recommendations.

Responsibility/accountability, transparency, and fairness are principles that have been previously reported as important in empirical work on public attitudes towards data intensive health technologies in Singapore (Ballantyne et al. 2022; Lysaght et al. 2020, 2021). In particular, the evidence suggests trustworthiness can be demonstrated where mechanisms for accountability and transparency are in place (Lysaght et al. 2020; Ballantyne et al. 2022). Our findings complement recent empirical bioethics research on AI acceptance in healthcare in other contexts. Shevtsova et al. (2024) identified numerous factors related to trust and acceptance of AI medical applications through a mixed-methods study across European countries. Their survey of healthcare stakeholders found that explainability, transparency, and physician oversight were among the most highly relevant factors influencing trust and acceptance of AI in medicine. Similarly, in a multinational study spanning 43 countries and surveying over 13,800 hospital patients, approximately 70% of patients preferred explainable AI with transparent decision-making processes, even if it meant compromising accuracy compared to black-box models. This strongly aligns with our finding that Singaporeans prioritise explainability and physician responsibility in AI-assisted healthcare decisions (Busch et al. 2025).

However, our findings are in contrast with research in the United States (Nussberger et al. 2022) where the performance of AI systems is prioritised over explainability. In that one study, participants rated interpretability as more important for high-stakes and resource-scarce decisions, such as medical care. However, when interpretability is traded off against AI accuracy, participants prioritise accuracy under the same high-stakes conditions. Other studies conducted in the US, the UK, Germany, Canada, and China have identified explainability (Turchioe et al. 2023; King et al. 2023), responsibility (Benda et al. 2024; Fritsch et al. 2022; Gould et al. 2023; Turchioe et al. 2023), and fairness (Lee and Rich 2021; Gao et al. 2020; Couture et al. 2023) as important values in medical AI. However, they did not carry out trade-off experiments to understand which principles are relatively more important than the others.

The significant association between respondents with a bachelor’s degree and the higher importance placed on responsibility in AI systems may be due to a more nuanced understanding of complex digital technologies and their implications, as well as greater exposure to their use. While more highly educated people are more likely to appreciate the potential benefits of AI, they are also more likely to advocate for the technology to be deployed ethically and responsibly, with doctors playing a vital role in the process. However, while higher education could be linked to higher technology literacy, it is not the only determinant. Therefore, we could not simply draw definitive conclusions from this finding, and further research is needed to explore this association.

The finding of a negative correlation between age and explainability preference suggests that younger respondents may place slightly higher value on understanding how AI systems work. Practically, this suggests that as Singapore’s population ages, maintaining strong explainability standards will remain important for building trust across generations, with potentially greater emphasis needed for younger patients who may have higher expectations for technological transparency.

The weak but significant positive correlation between the fear scale and quality preference indicates that respondents with higher technological anxiety may place greater emphasis on AI system performance, perhaps as a safeguard against perceived risks. This suggests that addressing performance concerns may be particularly important for building trust among those with greater AI apprehension. The general absence of statistically significant demographic differences across gender and ethnicity is itself a meaningful finding. This consistency implies that core values regarding AI governance (i.e., explainability, responsibility, and non-discrimination) are broadly shared across Singapore’s multicultural population. This contrasts with findings from many other contexts where demographic factors more strongly predict technology attitudes (Busch et al. 2025). The sociocultural context of Singapore may explain why small geographical size, high connectivity, and shared national narratives about technology and progress may create more unified perspectives on AI governance than might be found in larger, more regionally diverse countries.

Compared to the findings of Ploug et al. (2021), respondents in our study demonstrated relatively higher trust and higher levels of hope and fear in AI. This finding aligns with a 2023 international survey (Gillespie et al. 2023) about trust in AI where Singapore reported high levels of comfort, trust, and familiarity with the use of AI, adequacy of current AI regulation and governance, confidence in companies to use and govern AI, and the belief that AI will create jobs. Singaporeans are also more likely to report that AI will replace jobs. Additionally, 60% of Singaporeans reported high or complete confidence in the government to develop, use, and govern AI (Gillespie et al. 2023). While there is no comparable data on Danish citizens’ trust in AI, the 2022 OECD report indicated that 63% of the Danish population had confidence in their government (OECD 2023). In comparison, the 2023 Edelman Trust Barometer (Chew 2023) reported that 76% of respondents in Singapore trust their government.

Our findings were also similar to results from another 2024 global survey (2024) that spanned 32 countries. In this survey, 64% of Singaporeans agreed that AI makes them excited, while 56% reported nervousness. These percentages were higher than all European countries that participated in this survey, including Sweden, Italy, Switzerland, France, and the Netherlands. Singaporeans were also more inclined to report that AI has more benefits than drawbacks but also more likely to believe that AI will replace their current jobs in the next 5 years, compared to Europe. The high fear of surveillance, alongside significant hope for AI benefits, reflects Singapore’s relationship with technology governance. While Singapore has embraced digital solutions for public services and implemented comprehensive data collection systems (such as TraceTogether during the COVID-19 pandemic) (Chow et al. 2023), this has occurred alongside robust public discourse about privacy and data use (Das and Kwek 2024). The combination of high trust in government with concerns about surveillance suggests a sophisticated perspective that acknowledges both benefits and risks of AI. The results reflect Singapore’s pragmatic and forward-looking approach to new technologies (Detros 2024; Cheng 2024)—embracing beneficial innovations while remaining mindful of risks.

### Policy Implications for Implementation of Healthcare AI in Singapore

Our findings have policy implications for Singapore’s evolving healthcare AI governance framework. Based on our empirical findings, we recommend the following policy directions:

1. The AIHGle could incorporate tiered explainability standards based on clinical risk. For high-risk applications, AI systems should meet rigorous explainability benchmarks that enable physicians to understand and communicate recommendations to patients in laymen’s terms—reflecting the strong preference for AI systems whose suggestions can be explained like a doctor’s. Requiring the highest level of explainability across all healthcare AI applications would create innovation barriers and implementation challenges due to technical and resource constraints. A stratified approach balances the strong public preference for explainability with practical feasibility—ensuring rigorous standards for high-risk applications (high-risk treatment decisions) while allowing appropriate flexibility for lower-risk uses (such as low-stakes screening tools).
2. Singapore’s Smart Nation projects, including the National Electronic Health Record (NEHR) (Synapxe 2025) and HealthHub (https://www.healthhub.sg/), are increasingly incorporating AI technologies. Our findings suggest that as these platforms evolve, they should prioritise transparent explanation of how AI is used in personal health recommendations and maintain clear accountability structures.
3. The high value placed on non-discrimination testing suggests the need for Singapore to develop contextually appropriate fairness evaluation protocols that account for Singapore’s unique ethnic composition and healthcare access patterns. These protocols could consider incorporating factors such as
  Local demographic groups: testing AI systems specifically for performance equity across Singapore’s major ethnic groups,
  Socioeconomic dimensions: testing for potential biases related to housing type or socioeconomic status that might correlate with healthcare access patterns,
  Healthcare utilisation patterns: accounting for differences in how various communities access healthcare (polyclinics, private GPs, traditional medicine) that might influence training data representation,
  Intersectional analysis: assessing AI performance at the intersection of multiple demographic factors where small sample sizes might compromise algorithm performance.
4. The high levels of both hope and fear regarding AI suggest the need for national public education initiatives about healthcare AI that acknowledge both potential benefits and legitimate concerns. Such education programs could help the public understand the contexts in which AI generates healthcare recommendations, provide clarity about accountability frameworks in AI-assisted clinical decisions, address surveillance concerns through transparent explanation of data governance safeguards, and establish realistic expectations regarding AI capabilities and limitations.

These measures would align Singapore’s AI healthcare governance with public expectations while maintaining the country’s position as a regional leader in healthcare innovation.

## Limitations and Future Research

The respondents of this study are part of a survey panel and are likely more familiar with and interested in this topic as they have participated in previous surveys related to health and ethics. In addition, the choice-based conjoint methodology has inherent limitations for ethical trade-offs: (1) hypothetical bias may not capture real-world preferences; (2) preference stability cannot be assessed in our cross-sectional design; (3) pre-defined attributes may miss culturally specific concerns; and (4) cognitive complexity of trade-off tasks may lead to respondent simplification strategies. Lastly, while the attributes tested were considered important factors to AI decision-making in healthcare for the Danish population, they might not be directly applicable to Singapore.

Further research should aim to fine-tune how integrating AI into clinical decision-making practices can be done for the public good. This research could be done with additional empirical studies as well as deliberative methods of public engagement that can gain more nuanced understandings of community expectations and the ethical trade-offs they are willing to do to realise the potential benefits of AI. These studies could involve scenarios and vignettes to explore the trade-offs of different levels of AI explainability and responsibility with the practicality of AI implementation. Such studies could also aim to identify which values should be added, emphasised, or de-emphasised to best align with local cultural norms and values.

## Conclusion

This study provided fresh insights into what matters most to Singaporeans for the integration of AI decision-making in health care. Prior to advancing the technological quality of the AI system, there is a social and ethical obligation to first fulfil the following requirements: the AI system’s suggestions to be explainable like a doctor’s, subject to human doctors’ oversight, and rigorously tested to minimise discrimination.

## Data Availability

The data supporting the findings of this study are available upon reasonable request.
